# The effect of malaria rapid diagnostic tests results on antimicrobial prescription practices of health care workers in Burkina Faso

**DOI:** 10.1186/s12941-019-0304-2

**Published:** 2019-01-28

**Authors:** Massa dit Achille Bonko, Francois Kiemde, Marc Christian Tahita, Palpouguini Lompo, Athanase M. Some, Halidou Tinto, Michael Boele van Hensbroek, Petra F. Mens, Henk D. F. H. Schallig

**Affiliations:** 1 Institut de Recherche en Science de la Sante-Unite de Recherche Clinique de Nanoro, Nanoro, Burkina Faso; 2 Parasitology Unit, Department of Medical Microbiology, Amsterdam University Medical Centers, Academic Medical Centre, University of Amsterdam, Meibergdreef 9, 1105 AZ Amsterdam, The Netherlands; 3 Global Child Health Group, Amsterdam University Medical Centers, Academic Medical Centre, University of Amsterdam, Amsterdam, The Netherlands

**Keywords:** Prescription, Antimicrobial, Antibiotic, Fever, Malaria, Bacteria and parasites

## Abstract

**Background:**

Malaria rapid diagnostic tests (RDT) are widely used in endemic areas in order to comply with the recommendation that malaria treatment should only be given after the clinical diagnosis has been confirmed by RDT or microscopy. However, the overestimation of malaria infection with the use of *Pf*HRP2 based RDT, makes the management of febrile illnesses more challenging. This study aimed to assess the effect of the use of malaria RDT on antimicrobial prescription practices.

**Methods:**

A prospective study was conducted among febrile children under-5 years of age attending four health facilities and the referral hospital in the Nanoro Health District (Burkina Faso). To assess the effect of malaria RDT testing on the prescriptions of antimicrobials in febrile children, the initial diagnosis and antimicrobial prescriptions following a malaria RDT testing were recorded. The necessity of these prescriptions was subsequently checked by assessing the actual cause of fever by expert malaria microscopy and a microbiology analysis of blood, urine, stool and nasopharynx swabs that were collected from febrile cases to determine the actual cause of the fever episode.

**Results:**

Malaria was diagnosed by nurses, who are the primary health care providers, with a malaria RDT in 72.7% (798/1098) of febrile children, but only 53.7% (589/1097) cases could be confirmed by expert microscopy. Health care workers were likely to prescribe antimalarials to malaria positive RDT compared to malaria negative RDT (RR = 7.74, p = 0.00001). Malaria negative RDT result had a significant influence on the antibiotic prescriptions (RR = 3.57, p = 0.0001). The risk of prescribing antimicrobials was higher in health facility level compared to referral hospital. By cross-checking of laboratory findings to antimicrobial prescriptions, an important part of children with positive bacterial infection have received antibiotic prescriptions although the majority without any infection have also received antibiotics.

**Conclusion:**

Despite the good attitude of health care workers to adhere to diagnostic test results, antimalarials and antibiotics remain inappropriate prescribed to febrile children. The low specificity of malaria RDT used could be an important cause of these practices.

## Background

Acute febrile illnesses in children are globally one of the most common reasons to seek medical care and are associated with considerable morbidity and mortality [1, 2]. However, as these conditions often have no specific symptom(s), accurate diagnosis is difficult without laboratory facilities, which is commonly the case in many sub-Saharan countries (SSA) [2–6]. In the past, most febrile episodes encountered in these settings, were considered to be caused by malaria and were treated empirically with antimalarial drugs.

However, the recommendation by the World Health Organization (WHO) to confirm a malaria infection prior to treatment has resulted in the introduction of rapid diagnostic tests (RDT) and in a more focused prescription of antimalarial drugs [7–10]. Nevertheless there are nowadays indications that this positive development in the fight against malaria also has a downside due to an increased use of antibiotics in the malaria RDT negative population and this may contribute to the development of drug resistance [11, 12]. RDT’s to detect bacterial infections are not readily available in SSA and febrile patients have antibiotics prescribed almost at random in fear of missing a potentially life threatening infection. This situation becomes further complicated due to the fact that the most commonly used malaria RDT, based on *Plasmodium falciparum* histidine-rich protein-2 (*Pf*HRP2), have a specificity problem and may result in a significant number of false positive test results when compared with the golden standard, expert microscopy [13–16]. Consequently, this leads to inappropriate treatment with antimalarials too and the net result of the change from ‘presumptive’ to ‘RDT-*Pf*HRP2 based’ malaria management may not be the reduction in malaria over treatment as expected [11, 12, 17].

Moreover, the overestimation of malaria infection due to the use of *Pf*HRP2 based tests makes the management of febrile illnesses more challenging as the actual cause of fever could be missed. A previous study conducted in the same research area reported that probable alternative causes of fever were more prevalent in malaria RDT-*Pf*HRP2 false positive cases [18]. The screen and treat strategy for malaria might thus have created a diagnostic dilemma of “non-malaria infections” and how to manage them. Malaria RDT negative cases are treated with antibiotics regardless of the etiology of infection and all malaria false positive cases receive antimalarials unneeded. Consequently, the inappropriate use of antimalarials observed before the introduction of malaria RDT has now been replaced by an increased use of antibiotics in order to not miss potential treatable bacterial infections and of antimalarials due to false positive RDT.

In Burkina Faso, like in many other malaria-endemic areas, HRP2-based RDT is the test authorized by the Ministry of Health based on the specificity and sensitivity (> 95%) reported during the last WHO-FIND (World Health Organization—Foundation for Initiative New Diagnostics) to detect *Plasmodium falciparum* malaria infections [19]. However, the impact of the introduction of these RDT in clinical practice on the necessity of prescribing antimicrobials (antibiotics, antiparasitics and antimalarials) has not been evaluated. Therefore, in the present study this assessment was performed with the aim to get a better insight into the prescription practices in relation to malaria RDT test results that are available for the clinical staff that manage febrile patients.

## Materials and method

### Study site

This study was carried out in the health district of Nanoro, a rural area located around 85 km from Ouagadougou, the capital of Burkina Faso. Data collection was performed at four rural health facilities and the referral hospital (CMA Saint Camille de Nanoro) of Nanoro health district [18].

The peripheral health facilities are the first point of contact for guideline-based management of less complicated medical problems by nurses and for referral of more complicated cases [20]. Due to shortage of doctors in Burkina Faso, medical doctors are not available at this first level of the health care system. The referral hospital has medical doctors and well equipped laboratory facilities. Malaria is the first cause of consultation in children under-5 years of age in this region with a high transmission period during the rainy season between July and November [21].

### Study design and enrollment procedure

To investigate the effect of the use of a malaria RDT on the prescription of antimicrobials in the study area, a detailed descriptive study with a diagnostic component was performed.

The present study was conducted in the framework of a large survey to study the etiologies of fever episodes in children under-5 years of age in the Nanoro Health District [18, 22]. The whole population served by the 4 health centres is approximately 37,000 people and children under-5 years represent around 15% of this population (= around 5000 children). We assume that about half of these children (2500 children) will attend one of the health facilities because of fever related health problems. In order to have a representative sample of this population, we aimed to have around 1250 children included in this study (recruited over 1 year). Briefly, all children under-5 years of age with an axillary temperature ≥ 37.5 °C presenting at one of the participating health facilities or the referral hospital from January to December 2015 and from April to October 2016 were invited to participate in the study. Written informed consent was obtained from parents or legal guardians prior to any data or sample collection. A standard Case Record Form (CRF) was used to collect details on medical history (including prior drug prescription for current fever episodes) and clinical examination. Only the initial antimicrobial prescription made by the health care workers at the first contact with the participants was recorded. All participants were tested on site for malaria infection using a RDT-*Pf*HRP2 supplied the Ministry of Health and a primary diagnosis was made by the health facility nurses and this was recorded on the CRF.

The performance of malaria RDT supplied by the Ministry of Health, and used in the present research, has been previously assessed in the study area [18, 23]. The clinical diagnosis by the nurse was made using the International Classification of Diseases (9th version) [24]. The participants were managed according to the Burkinabe guideline based on the WHO guideline for the integrative management of childhood illness [20].

Additional clinical specimens, i.e. blood, stool, urine and nasopharyngeal swabs, were collected for microbiology analyses and malaria microscopy (blood sample only) at the laboratory of the Clinical Research Unit of Nanoro (CRUN). Parents or guardians were asked to complete the urine and/or stool sample collection at a later stage when initial sample collection could not be completed during the enrolment procedure. When a positive culture or test justified an additional or alternative treatment, the results were communicated to the nurses in the respective health facility and if needed additional treatment was provided to the patient as soon as possible.

This study was approved by the National Ethical Committee in Health Research, Burkina Faso (Deliberation N°2014-11-130).

### Laboratory procedures

All laboratory procedures employed in this study have been described in detail previously [22]. In brief, the following procedures were performed:

Malaria RDT were performed according to the manufacturer’s instructions by nurses for each febrile child attending the health facilities that were used as a recruitment site. The malaria RDT used is the *Pf*HRP2 detecting SD Bioline *Pf* test (Standard Diagnostics, Hagal-Dong, Korea) supplied by the Burkinabe National Malaria Control Program (Ministry of Health). The results of malaria RDT testing were recorded as positive or negative.

Expert malaria microscopy on thin and thick blood smears was performed by two independent expert microscopists and in case of discordance (positive vs. negative, difference in *Plasmodium* species, difference in parasite density Log [difference] > Log10 or ratio > 2 in case of parasite density ≤ 400/µl and > 400/µl respectively), a decisive reading was performed by a third expert.

Blood cultures were done using 1–3 ml of venous blood collected in pediatric blood culture bottles (BD BACTEC Peds Plus™/F, Becton–Dickinson, and Company, Sparks, Maryland, USA), incubated in a BACTEC 9050 instrument (Becton–Dickinson) for a total of 5 days. In case of a positive growth signal a Gram straining was done and subsequent culture was performed at 35–37 °C for an additional 18–24 h.

Stool cultures were performed on Eosin-Methylene Blue (EMB) agar (for children less than 2 years of age), Hektoen agar and inoculated in selenite of sodium broth. The culture media were incubated at 35–37 °C for 18–24 h, and the selenite broth at 35–37 °C for 4 h before putting in culture on *Salmonella* and *Shigella* agar (*SS* agar). In addition, SD Bioline Rota/Adeno RDT (Standard Diagnostics, Inc., Korea) was used to detect Group A rotavirus or adenovirus serotype 40/41 in stool samples. Finally, fresh stool samples were used for parasitological examination for the presence of cysts, eggs, and vegetative forms of protozoa by microscopy. In case of suspicion of cysts, a second slide was prepared using lugol for confirmation.

Urine samples were tested with a dipstick (Standard Diagnostics, UroColor, Inc) for the presence of leucocytes and nitrite. If positives for these parameters, a culture of the sample was done on Cystine Lactose Electrolyte Deficient (CLED) and EMB agar and incubated at 35–37 °C for 24 h.

Nasopharyngeal swabs were collected from each participant, placed in skim milk–tryptone–glucose–glycerol (STGG) broth and transported to the central CRUN laboratory in a dark storage box at room temperature. For analysis, 200 μl of this broth was vortexed and introduced in 5 ml of Todd-Hewitt (TH) broth for enrichment and incubated in two steps (before and after plating) for two times 24 h to detect *Streptococcus pneumoniae* and *Staphylococcus aureus* species (*S. pneumoniae* and *S. aureus* can be a cause of fever, but may also be carried in the nasopharynx of healthy people). Bacterial isolates from all cultures were identified by standard microbiological methods [25] and/or by Analytical Profile Index (API) biochemical test kit (bioMerieux, France).

### Data analysis

Data were double entered using OpenClinica software. Description of qualitative and quantitative variables was performed using proportion. To assess the effect of malaria RDT on antimicrobial prescriptions, only the first antimicrobial prescription provided by the health care workers after obtaining the results of the malaria RDT was recorded. The effect of malaria RDT on the prescription of antimicrobials was assessed by cross-checking the prescription done based on malaria RDT results and malaria microscopy performed by expert microscopist. The risk ratio of a febrile child tested with malaria RDT (positive versus negative) to receive an antimicrobial prescription (i.e. antimalarial, antibiotic and antiparasitic) compared to the outcome of expert malaria microscopy was calculated using the binomial regression with a log link. The risk ratio of each antimicrobial prescribed at health facilities and referral hospital based on malaria RDT results was also calculated. Data analysis was done using STATA 13 software package^®^ and a p-value less than 0.05 was considered statistically significant.

## Results

### Demographic characteristics of the study population

A total of 1099 children were included in the study. During the analysis it became apparent that information on one malaria RDT result and two malaria slide readings were missing and these were considered as missing data for the analysis. Malaria was the most frequently infection diagnosed by nurses by malaria RDT in 72.68% (798/1098) of febrile children, but only 53.69% (589/1097) of febrile children could be confirmed by expert microscopy. The second commonest cause of fever diagnosed by heath facilities nurses were respiratory tract infections (RTI) [bronchiolitis 9.2%(101/1099), pneumonia 14.47% (159/1099), other RTI 14.10% (155/1099)]. The characteristics of study population are presented in Table 1.

**Table 1:** Basic characteristic of the study population comprising of 1099 children under-5 years of age with fever (axillary temperature ≥ 37.5 °C)

	No (%)
Sex	
Male	607 (55.23)
Age	
≤ 12 months	306 (27.84)
Recruitment site	
Referral hospital	294 (26.75)
Health facilities	805 (73.25)
Clinical diagnosis (n = 1099)^a^	
Malaria based on malaria RDT^b^	798 (72.68)
Septicaemia	2 (0.18)
Gastro-enteritis	268 (24.39)
Malnourished	33 (3.00)
Bronchiolitis	101 (9.20)
Pneumonia	159 (14.47)
Other GII	56 (5.10)
Other RTI	155 (14.10)
Urinary tract infection	15 (1.36)
Laboratory findings	
Malaria based on microscopy (n = 1097)^c^	589 (53.69)
Bacterial bloodstream infection (n = 1099)	65 (5.91)
Parasitic gastro-intestinal infection (n = 757)	215 (28.40)
Bacterial gastro-intestinal infection (n = 757)	65 (8.59)
Viral gastro-intestinal infection (n = 757)	29 (3.83)
Urinary tract infection (n = 739)	11 (1.49)
Common bacterial pathogens of nasopharynx (n = 629)	153 (24.32)

*RDT* rapid diagnostic test, *GII* gastro-intestinal tract infection, *RTI* respiratory tract infections

^a^Based on RDT testing and clinical assessment by attending Health Worker

^b^RDT was not performed for one child N = 1098)

^c^Two malaria slides were not performed

A small percentage of the febrile children who received antimalarial prescription got first an injectable antimalarial (artesunate or artemether) followed by artemisinin-based combination therapy (artemether–lumefantrine or artesunate–amodiaquine) for subsequent home treatment 2.11% (17/805). The majority of malaria treatment 97.88% (788/805) was artemisinin-based combination therapy as previously mentioned. For the antibiotic prescriptions, 14.58% (125/857) and 1.17% (10/857) have received respectively 2 and 3 antibiotic prescriptions, and 84.24% (722/857) received a single prescription. All antiparasitics were single prescriptions (Table 2).

**Table 2:** Distribution of antimicrobial prescription among febrile children

Number of antimicrobial(s) prescribed	Number of children who received antimicrobial prescriptions		
	Antibiotics (n = 857) % (n/N)	Antimalarials (n = 805) % (n/N)	Antiparasitics (n = 197) % (n/N)
1 prescribed	84.25 (722/857)	97.88 (788/805)	100 (197/197)
2 prescribed	14.58 (125/857)	2.12 (17/805)	–
3 prescribed	1.17 (10/857)	–	–

### Influence of malaria PfHRP2-RDT on antimicrobial prescriptions

Table 3 presents the risk of antimicrobial prescriptions according to malaria RDT results during the study period. It is evident that rural health facilities as well as the referral hospital were likely to prescribe an antimalarial in case of a positive malaria RDT, as recommended by WHO, compared to negative tested cases. Antibiotics were likely to be prescribed to negative malaria RDT. The adherence rate of the health care workers to the result of the malaria RDT-*Pf*HRP2 was 92.89% (1020/1098: 762 malaria RDT positive patients received an antimalarial treatment and 258 malaria RDT negative cases did not receive an antimalarial treatment). However, if malaria expert microscopy is considered as gold standard, the risk for febrile children tested with RDT-*Pf*HRP2 to have their initial antimalarial prescription affected (modified) was statistically significant (RR = 7.74, p = 0.00001). Moreover, the likelihood of antibiotic prescription in case of a negative malaria RDTs was 3 times higher compared to positive malaria RDTs and statistically significant (RR = 3.57, p < 0.0001).

**Table 3:** Antimicrobial prescriptions according to malaria test results using malaria RDTs

Percentage of antimicrobial prescribe	Children tested positive for malaria infection by microscopy and RDT		Children tested negative for malaria infection by microscopy and RDT		Risk ratio of antimicrobial prescription for malaria RDT (95% CI)	p-value
	RDT positive, (n = 798)^a^	Microscopy positive, (n = 589)^b^	RDT negative, (n = 300)^a^	Microscopy negative, (n = 508)^b^		
Antimalarial	762 (95.48)	564 (95.75)	42 (14)	239 (47.04)	7.74 (5.69–10.51)	< 0.00001
Antibiotic	578 (72.43)	394 (66.89)	278 (92.66)	462 (90.94)	3.57 (2.37–5.38)	< 0.00001
Antiparasitic	118 (14.78)	93 (15.78)	53 (17.66)	78 (15.35)	1.16 (0.90–1.49)	0.240

^a^RDT was not performed in one child

^b^Malaria microscopy was not performed in two children

It is apparent from Table 4 that the health care workers at the rural health facilities were more likely to prescribe antimicrobials to children who tested positive for malaria by RDT (antimalarial = 96.11%; antibiotics = 75.20%; antiparasitics = 18.63%) than at the level of the referral hospital (antimalarial = 93.37%; antibiotics = 62.98%; antiparasitics = 1.65%). Furthermore, the nurses in the rural health facilities were more likely to prescribe antibiotics (97.86%) and antiparasitics (26.73%) to children who tested negative by malaria RDT than attending health staff at the referral hospital (antimalarial = 84.07%; antiparasitics = 2.65%), except for malaria treatments (rural health facilities = 7.48%; referral hospital = 24.77%). As an overall trend it was found that the risk of prescribing antimalarial as well as antibiotic and antiparasitic to children with a positive malaria RDT compared to children with negative malaria RDT was higher in health facility compared to referral hospital. The risk of prescribing antimalarial in positive tested patients compared to negative malaria RDTs was 8.01 (95% CI 5.51–11.66, p = 0.00001) and 6.93 (95% CI 4.07–11.81, p = 0.00001) for the rural health facility and referral hospital, respectively. The risk of prescribing antibiotic in case of negative malaria RDT compared to RDT positive was 11.10 (95% CI 4.18–29.43, p = 0.00001) in health facility and 2.14 (95% CI 1.38–3.32, p = 0.00001) in referral hospital.

**Table 4:** Antimicrobial prescriptions done at the of site recruitment (heath facility and referral hospital)

Antimicrobial	Children tested positive for malaria RDT		Children tested negative for malaria RDT		Risk ratio for antimicrobial prescription for health facilities patient		Risk ratio for antimicrobial prescription for referral hospital	
	Health facility level, (n = 617)	Referral hospital, (n = 181)	Health facility level, (n = 187)	Referral hospital, (n = 113)	RR (95% CI)	p-value	RR (95% CI)	p-value
Antimalarial	593 (96.11)	169 (93.37)	14 (7.48)	28 (24.77)	8.01 (5.51–11.66)	< 0.00001	6.93 (4.07–11.81)	< 0.00001
Antibiotic	464 (75.20)	114 (62.98)	183 (97.86)	95 (84.07)	11.10 (4.18–29.43)	< 0.00001	2.14 (1.38–3.32)	< 0.00001
Antiparasitic	115 (18.63)	3 (1.65)	50 (26.73)	3 (2.65)	1.41 (1.07–1.86)	0.0163	1.30 (0.58–2.95)	0.556

### The limitations of empiric antimicrobial prescriptions by health workers

By cross-checking the laboratory findings (actual cause of disease) with the antimicrobials prescribed by health care workers based on the routine practice (based on the national guideline for the treatment of childhood diseases), it is evident that a large part of the febrile children who received an antibiotic prescription did actually not need such a treatment. It was that at the rural health facilities all children with a positive bacterial bloodstream infection (bBSI) (25/25) or urinary tract infection (UTI) (8/8) and 80.39% (41/51) with bacterial gastro-intestinal infection (bGII) based to laboratory results did actually receive antibiotic prescriptions. But also 93.98% of the febrile children without any infection (confirmed by laboratory testing) in the present study too actually received antibiotic prescriptions. In contrast, at the referral hospital only 75% (30/40) of children with positive bBSI, 64.28% (9/14) with bGII and 100% (3/3) with UTI did actually receive antibiotic prescription. Moreover, 86.04% (74/86) of febrile children without (laboratory confirmed) infection did also got antibiotic prescriptions (Table 5).

**Table 5:** Antibiotic and antiparasitic prescriptions at the referral hospital and health facilities according to laboratory findings

	bBSI (n = 65) n/N (%)	bGII (n = 65) n/N (%)	pGII (n = 215) n/N (%)	vGII (n = 29) n/N (%)	UTI (n = 11) n/N (%)	CBPN (n = 153) n/N (%)	No infection^a^ (n = 269) n/N (%)
Referral hospital	40/65 (61.54%)	14/65 (21.54)	40/215 (18.60)	6/29 (20.69)	3/11 (27.27)	41/153 (26.80)	86/269 (31.97)
Antimalarial	31/40 (77.5)	8/14 (57.14)	29/40 (72.5)	3/6 (50)	3/3 (100)	21/41 (51.21)	30/86 (34.88)
Antibiotic	30/40 (75)	9/14 (64.28)	29/40 (72.5)	6/6 (100)	3/3 (100)	33/41 (80.48)	74/86 (86.04)
Antiparasitic	0/40 (0)	0/14 (0)	1/40 (2.50)	1/6 (16.67)	0/3 (0)	1/41 (2.43)	2/86 (2.32)
Health facilities	25/65 (38.46)	51/56 (78.46)	175/215 (81.40)	23/29 (79.31)	8/11 (72.73)	112/153 (73.20)	183/269 (68.02)
Antimalarial	16/25 (64)	39/51 (76.47)	129/175 (73.71)	13/23 (56.52)	7/8 (87.5)	85/112 (75.89)	86/183 (46.99)
Antibiotic	25/25 (100)	41/51 (80.39)	141/175 (80.57)	20/23 (86.95)	8/8 (100)	97/112 (86.60)	172/183 (93.98)
Antiparasitic	5/25 (20.00)	16/51 (31.37)	30/175 (17.14)	4/23 (17.39)	1/8 (12.5)	20/112 (17.85)	47/183 (25.68)

*bBSI* bacterial bloodstream infection, *bGII* bacterial gastro-intestinal infection, *vGII* viral gastro-intestinal infection, *UTI* urinary tract infection, *CBPN* common bacteria pathogens of nasopharynx

^a^Including malaria infection

## Discussion

A rapid diagnostic test based on the detection of *P. falciparum* specific HRP-2 antigen is one of the few diagnostic tools available for health care workers in many resource limited settings in malaria endemic settings to assist them in differentiating between malaria fever and other causes of fever in many malaria. As previously reported, HRP-2 based RDTs displayed a low specificity (59%) in the current study area [18]. This low specificity of HRP2 based RDT was also reported in other malaria endemic areas [13, 26–28]. Therefore, expert microscopy was performed in the present study in the central laboratory of CRUN to further determine (by cross-checking) whether antimicrobials were appropriately prescribed by the attending health care staff. Despite the good attitude of health care workers to nearly always use malaria RDT in case of a febrile patient and act accordingly to the result of diagnostic testing (92.89 of adherence of health care workers to malaria RDT), the prescription of antimalarials is affected by the performance of the malaria RDT used in the study area [23]. Due to the persistence of HRP2 antigen after successful antimalarial treatment, malaria infection is overestimated (as a result of a false positive RDT) leading to an inappropriate prescription of antimalarials [13–16]. Furthermore, even if the diagnosis malaria was established by RDT, there was a tendency to prescribe antibiotics next to antimalarials too. Moreover, and even worse, the prescription of antibiotics becomes almost systematic when a malaria infection could be excluded. The majority of outpatients were visiting the rural health facilities where there is a lack of laboratory facilities that can confirm the actual cause of infection and this explains the inappropriate prescription of antibiotics at this level. In contrast, at the level of the referral hospital these facilities are available, but there the attending hospital health care workers might not feel confident to postpone antibiotic treatments in febrile children until the microbiology results become available in fear of treating a potentially life threating, but treatable, bacterial infection too late or overlooking such an infection. Given the fact that health care workers tend to adhere well to malaria RDT results as reported in our study area [this study and previous study by Ruizendaal et al. [29], it is most probable that health system may be able to deal with the problem of inappropriate prescription of antibiotics as long as the health care workers get the appropriate diagnostic tools. Therefore, the development of (near) point-of-care tests to screen for other causes of fever becomes essential to guide appropriate antibiotic prescriptions.

The inappropriate prescription of antibiotics and antimalarial treatments observed in the present study could be a consequence of the rightful fear of health care workers to miss a treatable bacterial infection. Indeed, a significant number of potentially treatable etiologies were missed in the present study and others [18, 30]. Despite the tendency of systematic prescription of antibiotics to febrile children, an important number of children with bacterial infections did not receive appropriate treatment mainly at the referral hospital at the first contact compared to the rural health facilities. This observation can be explained by the availability at referral hospital of equipped laboratory allowing for a better diagnostic compared to health facilities where malaria RDT is the only diagnostic tool available. Moreover, in Burkina Faso, nurses working at health facilities are responsible for the primary management of patients, while at the referral hospital there are medical doctors who are better trained to make a specific prescription. The fear to overlook a potential bacterial infection by sending a sick child back home without prescribing medication leads to the antibiotic prescriptions to an important part of febrile children without bacterial infections. This tendency of systematic prescription of antibiotics to febrile children is considered in many setting to be more a social and behavioral issue than a medical problem [31]. To tackle this problem, there is a need to evaluate and understand the context in which the (over) prescriptions occur. Moreover, more diagnostic tools should become available that can be implemented in resource limited settings that can aid proper antibiotics prescriptions and thereby reducing the risk of inappropriate prescription and of emerging of antibiotic resistance.

Antiparasitics drugs were not much prescribed in this study. Based on the laboratory findings, there was a high under-prescription of antiparasitics as actually a significant number of febrile children who actually had gastro-intestinal parasites did not receive appropriate treatment. Most likely, the more obscure symptoms associated with a parasitic gastro-intestinal infection, which in most cases do not cause fever, are probably overlooked in favor of other febrile etiologies that are more readily diagnosed.

Despite the recommendation of the WHO to treat all children with a positive malaria RDT or microscopy result, few cases (4.52%) of febrile children who had a malaria positive RDT did not receive antimalarials. The reason for this non adherence was not assessed in the present study. Possibly, complementary information on previous drug use provided by the parents/guardians to the attending nurses suggested that these children did not require antimalarial prescriptions and that the RDT were positive in these cases was interpreted as a consequence of persisting HRP2 antigen (up to 4 weeks or even more) after successful treatment for malaria [13–16]. In contrast, 14% of the children with a malaria RDT negative result still received antimalarial prescriptions. It has not been determined in the present study what the rationale was behind this non-compliance. This lack of adherence to the protocols in place for the management of malaria has also been reported elsewhere [32]. Therefore, it is of utmost importance to further study the motivation of health care workers to not adhere to this protocol recommended by the WHO and the National Malaria Control Program (NMCP) and this should preferably be done, in combination with to further training and education of the health practitioners.

A possible limitation of our study could be the lack of confirmation of pneumonia cases presumptively diagnosed by health care workers. Although the nasopharynx of some children was colonized by common bacterial pathogens, such as *Staphylococcus aureus* and *Streptococcus pneumoniae*, a relation of their presence with ongoing fever has not been established [22]. Therefore, it might be possible that some of these children did not have pneumonia. Moreover, RTI are the second cause of fever diagnosed presumptively by health facility nurses after malaria in the present study. The WHO guideline recommends the prescription of antibiotics to children with (suspected) pneumonia but not to children with acute bronchitis [1, 20, 33, 34]. In the present study, a radiological confirmation of pneumonia could not be done in children suspected of RTI, because this diagnostic technology is was not available at the participating health centres. Another possible limitation of the present research is the potential under-diagnosis of the number of UTI and GII cases as some urine and stool samples were not collected during recruitment. Finally, viral etiologies are known to be responsible for a significant number of fever episodes in children [17, 35, 36], but these were not studied in the present study as the CRUN laboratory does not have facilities to perform virus identification. Although viral febrile infections do not need antibiotic treatment, they can also be a cause of over prescriptions as there is fear of overlooking potential bacterial infections. A rapid diagnostic test (or combination of tests) that can broadly differentiate between bacterial and viral infections would be very helpful in this respect [37–39].

## Conclusion

Despite the correct attitude of health care workers to treat malaria according to a diagnostic test result, antimalarials and antibiotics are still inappropriately prescribed by nurses. The low specificity of the malaria RDT used and the absence of practical tools to diagnose bacterial infections could be important causes of this inadequate prescription practice. In addition, the fear to delay antibiotic treatment or to overlook a treatable bacterial infection, leads to the inappropriate prescription of antibiotics. The health system is likely to deal with the problem of inappropriate prescription if appropriate diagnostic tools are developed and implemented.

## Abbreviations

API: Analytical Profile Index
bBSI: bacterial bloodstream infection
CMA: Centre Medical avec Antenne Churigicale
bGII: bacterial gastro-intestinal infection
CI: confidence interval
CLED: cystine lactose electrolyte deficient
CRF: case report form
CRUN: Clinical Research Unit of Nanoro
EMB: eosin-methylene blue
FIND: foundation for initiative new diagnostics
HRP-2: histidine-rich protein-2
NMCP: National Malaria Control Program
*Pf*: *Plasmodium falciparum*
RDT: rapid diagnostic test
RR: risk ratio
RTI: respiratory tract infections
*S. aureus*: *Staphylococcus aureus*
*S. pneumoniae*: *Streptococcus pneumoniae*
SD: standard diagnostic
*SS*: *Salmonella* and *Shigella*
STGG: skim milk–tryptone–glucose–glycerol
TH: Todd-Hewitt
UTI: urinary tract infection
WHO: World Health Organization
